# Supplementary material for: TORC2 inactivation promotes heterochromatin formation in rDNA and prolongs viability of quiescent fission yeast cells
Source: Commun Biol. 2025 Nov 19;8:1606. doi: 10.1038/s42003-025-08953-5 (PMC12630781; doi:10.1038/s42003-025-08953-5)
Supplement: Supplementary file 5 — Supplementary Information [file 42003_2025_8953_MOESM5_ESM.pdf]

Supplementary Information

TORC2 inactivation promotes heterochromatin formation in rDNA and prolongs viability of quiescent fission yeast cells

Hayato Hirai and Kunihiro Ohta

Supplementary tables 1 and 2
Supplementary figures 1–5

Supplementary table 1. *S. pombe* strains used in this study

Strain	Genotype	Source	Figures
975	h^{+}	Our stock	1b, e, f, 2a-c, 3a, e-g, 4c-e, 5a-e, S2b, S4a, b, S5
HH589	h^{+} *tor1-FLAG-kan*	This study	1b, c, S1
HH315	h^{+} *tor1::hph*	This study	1e, f, 2a-c, 5a-f, S2b, S4a, S5a-c
HH553	h^{+} *sin1::kan*	This study	1e
HH555	h^{+} *wat1::kan*	This study	1e
HH558	h^{+} *ste20::hph*	This study	1e
HH582	h^{+} *GFP-swi6-kan gar2-mCherry-nat*	This study	2d-h
HH588	h^{+} *GFP-swi6-kan tor1::hph gar2-mCherry-nat*	This study	2d-h
HH537	h^{+} *gad8-FLAG-kan*	This study	3a-d, S3
HH584	h^{+} *gad8-FLAG-kan tor1::hph*	This study	3c, d, S3b
HH567	h^{-} *gad8::hph*	This study	3e-g, S4b
HH591	h^{+} *leo1-FLAG-kan*	This study	4a
HH592	h^{+} *leo1-FLAG-kan*	This study	4b
HH593	h^{+} *leo1-FLAG-kan gad8::hph*	This study	4b
HH595	h^{+} *leo1::hph*	This study	4c-e
HH587	h^{+} *clr4::kan tor1::hph*	This study	5f, S5c
HH21	h^{+} *gcn5-3HA-kan*	Our stock	S4c
HH576	h^{+} *gcn5-3HA-kan gad8::hph*	This study	S4c
FY33858	h^{-} *tor2-287*	NBRP	S5a, b
HH529	h^{-} *tor2-287 tor1::hph*	This study	S5a, b
HH202	h^{+} *clr4::kan*	This study	S5c, d

Supplementary table 2. Primers used in this study

Name	Direction	Sequence
rDNA_I	Fw	TGAAGCGGGTGTAAAGATGAG
rDNA_I	Rv	GAATGACTGTTTTTGCATTCCAG
rDNA_II	Fw	CACCGAAATGGACGAAATTCAC
rDNA_II	Rv	CCTTGGACCTATTTTTCCTCGA
rDNA_III (18S)	Fw	TCGGCACCTTACGAGAAATC
rDNA_III (18S)	Rv	TATGTCTGGACCTGGTGAGT
rDNA_IV (5.8S)	Fw	CAACGGATCTCTTGGCTCTC
rDNA_IV (5.8S)	Rv	CGATGATTCACGGAATTCTGCA
rDNA_V (28S)	Fw	GAGACCGATAGCGAACAAGTAG
rDNA_V (28S)	Rv	TGACCTACCACAGGTTCAGAG
rDNA_VI	Fw	AGAAAAAGTCGAGCGAGTCG
rDNA_VI	Rv	TCTTCAAAAGTGCATTACCCTTAC
ITS2	Fw	TTTTTGATGAGGTGTTGAACGA
ITS2	Rv	ACAAAGTGGTAAAACCTATTACGTTC
5'ETS	Fw	TCTACGTGACGGTGCTTGAC
5'ETS	Rv	ACTCATCGAGTCTCCACAAGAC
prp3	Fw	GCACAGTCGTTGTACAAATTCGTATTCCC
prp3	Rv	ACGATTCTAAACGCCTCTTGTTACGATCC
act1	Fw	CTCTGGTGATGGTGTTACCCACACTG
act1	Rv	AAGTCACGACCGGCGAGATCAAG
act1 (RT-qPCR)	Fw	TACCCCATTGAGCACGGTAT
act1 (RT-qPCR)	Rv	CTTCTCACGGTTGGATTTGG

Supplementary figures

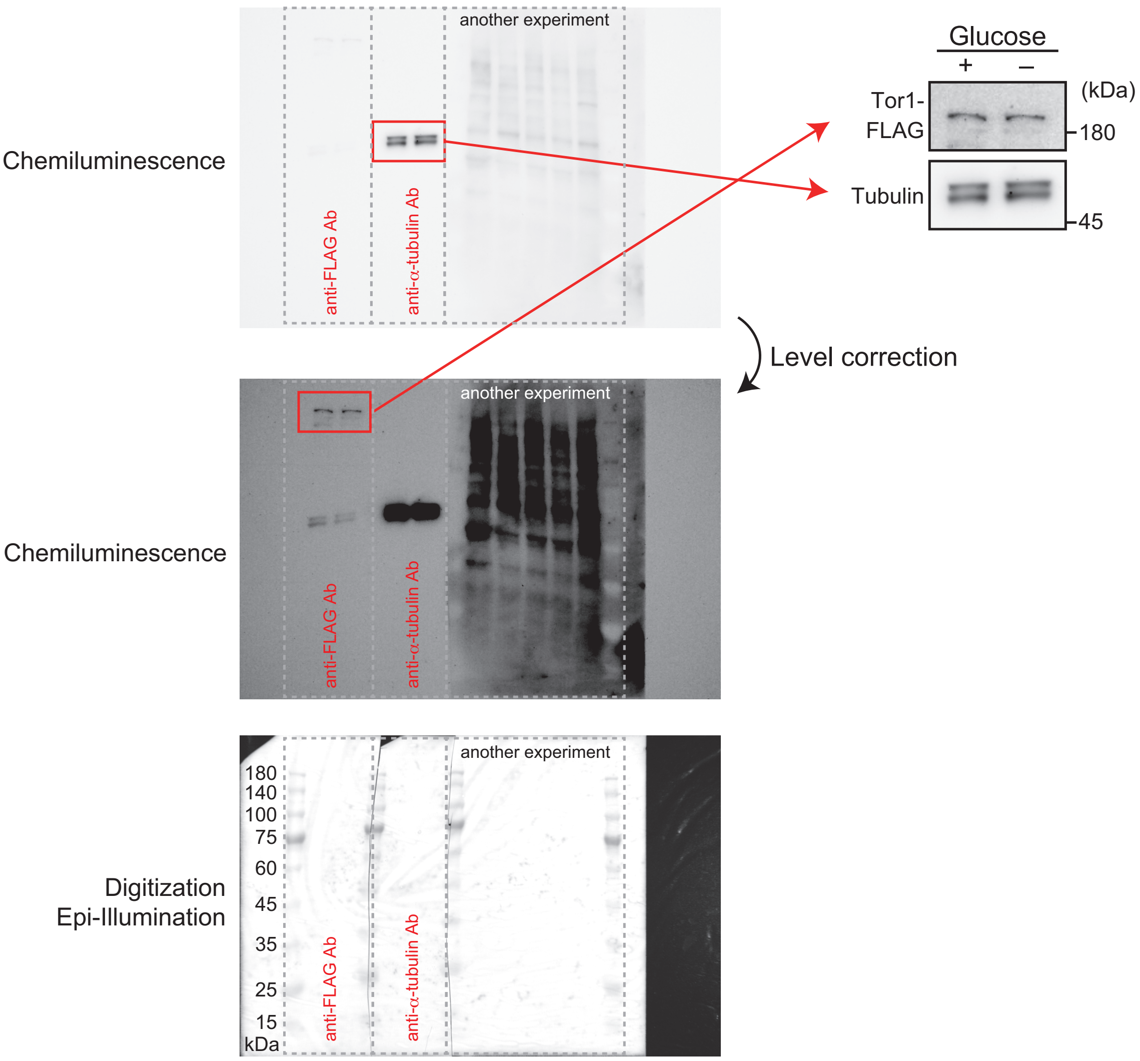

Supplementary Fig. 1 The original, uncropped image for Fig. 1c

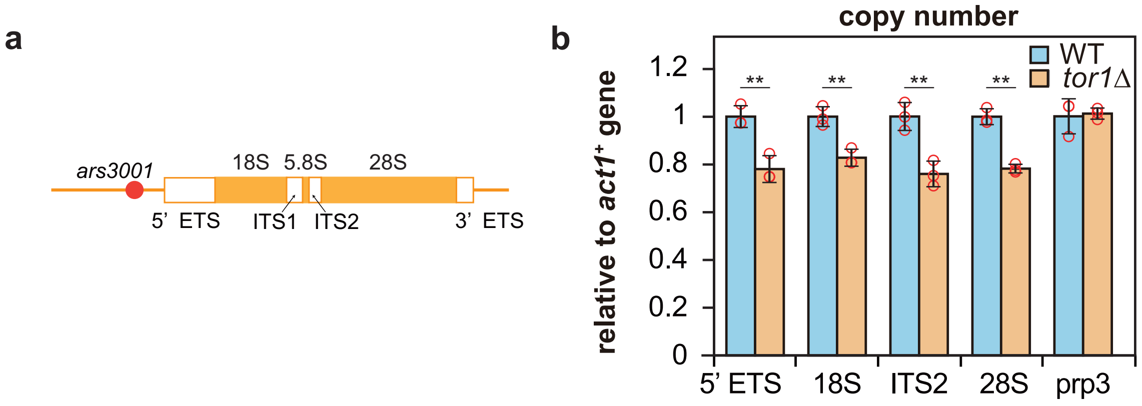

Supplementary Fig. 2 rDNA copy number slightly decreased in the *tor1Δ* mutant compared to the wild-type

(a) Schematic diagram of the rDNA region in fission yeast. **(b)** Copy number of each rDNA locus normalized to the *act1* gene. The *tor1Δ* strain exhibited a ~20% reduction in rDNA copy number, but showed no change in the *prp3* gene. Data are presented as mean ±SD, n = 3 biological replicates. *p* values were calculated by Student's *t*-test. ***$p < 0.001$, **$p < 0.01$, *$p < 0.05$.

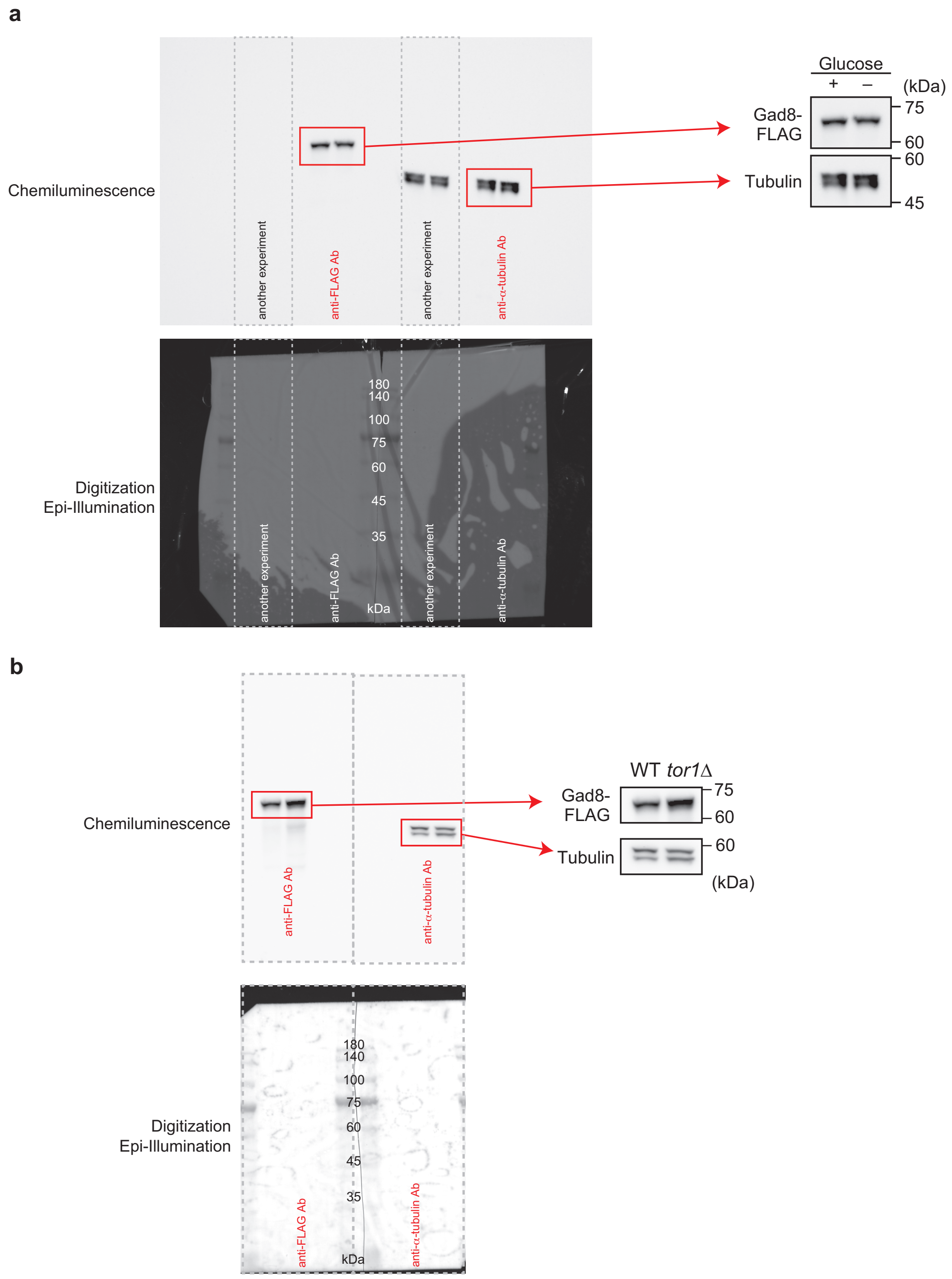

Supplementary Fig. 3 The original, uncropped image for Fig. 3b and c

(a) Uncropped Western blot images of Gad8-FLAG and Tubulin used in Fig. 3b.

(b) Uncropped Western blot images of Gad8-FLAG and Tubulin used in Fig. 3c.

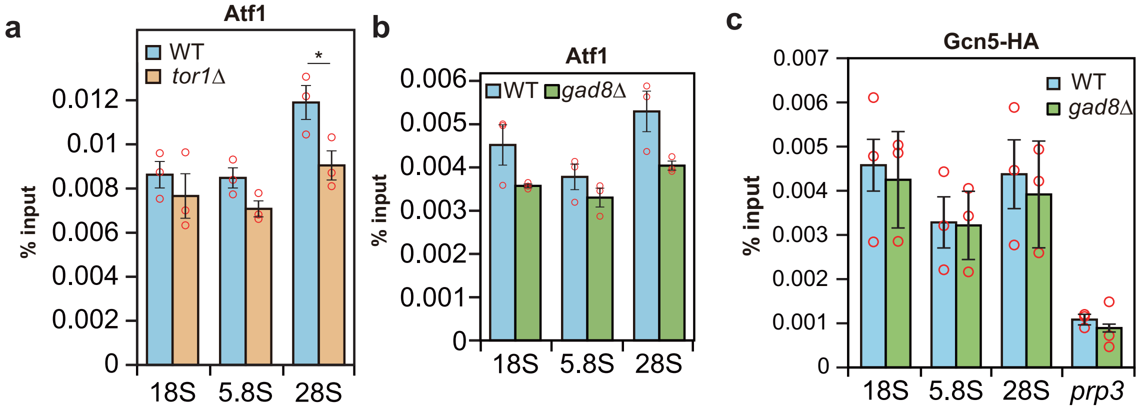

Supplementary Fig. 4 The localization of Atf1 and Gcn5 in the rDNA remained consistent across WT, *tor1*Δ, and *gad8*Δ cells

(a, b) ChIP-qPCR data showing Atf1 enrichment in the rDNA region in WT, *tor1*Δ **(a)**, and *gad8*Δ **(b)** cells. Data are presented as mean ±SEM, n = 3 biological replicates. **(c)** IP/input ratios for Gcn5-HA at 18S, 5.8S, and 28S regions of rDNA as well as the *prp3* gene in WT and the *gad8*Δ mutant. Data are presented as mean ±SEM, n = 3 biological replicates.

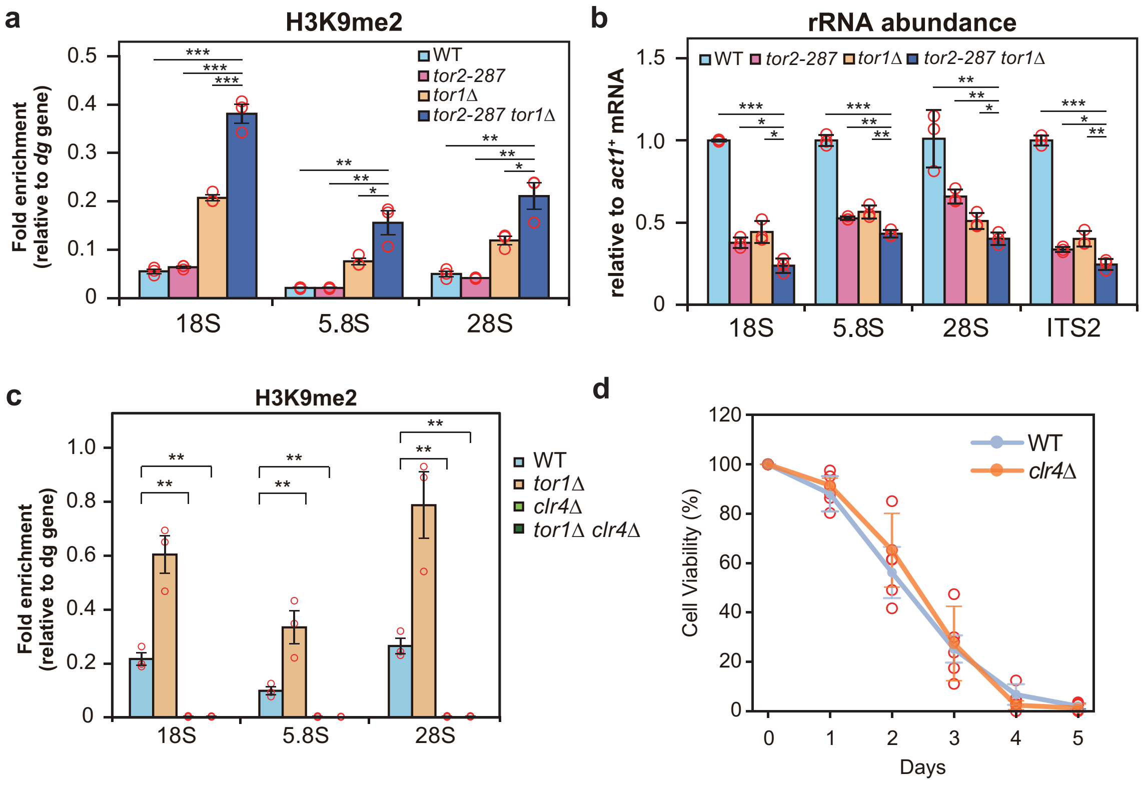

Supplementary Fig. 5 Severe rDNA heterochromatin formation with reduced rRNA levels was observed in the *tor2-287 tor1*Δ double mutant

(a) ChIP-qPCR data showing H3K9me2 levels in the rDNA normalized to the pericentromeric region (dg) in WT, *tor2-287*, *tor1*Δ, and *tor2-287 tor1*Δ cells. Data are presented as mean ±SEM, n = 3 biological replicates. **(b)** rRNA levels normalized to *act1* mRNA in WT, *tor2-287*, *tor1*Δ, and *tor2-287 tor1*Δ cells. Data are presented as mean ±SD, n = 3 biological replicates. **(c)** H3K9me2 levels in the rDNA relative to the pericentromeric region (dg) in WT, *tor1*Δ, *clr4*Δ, and tor1Δ *clr4*Δ cells. Data are presented as mean ±SEM, n = 3 biological replicates. (d) Graph showing chronological lifespan in WT and *clr4*Δ cells. Data are presented as mean ±SD, n = 3 biological replicates. *p*-values were calculated by Student's *t*-test. ***$p < 0.001$, **$p < 0.01$, *$p < 0.05$.
